# Fine mapping and transcriptomics reveal *OSG* function in regulation of grain size and pollen fertility in rice (*Oryza sativa*)

**DOI:** 10.1371/journal.pone.0338401

**Published:** 2026-01-08

**Authors:** Sijie Zhao, Siyue Zhang, Weichen Xu, Sijia Fang, Mei Liu, Lei Wang, Xianyang Zhang, Bingxu Chen, Shuya Wei, Heming Zhao

**Affiliations:** 1 Center for Crop Biotechnology, College of Agriculture, Anhui Science and Technology University, Chuzhou, China; 2 College of Bioengineering, Wuhan Technical University, Wuhan, China; Government College University Faisalabad, PAKISTAN

## Abstract

Grain shape is a critical factor that directly influences rice yield and quality, however, the molecular mechanisms underlying the regulation of grain shape development remains elusive. In this study, we characterized an *oval-shaped grain* mutant, *osg*, from the rice radiation mutagenesis mutant library via ^60^Co-γ ray irradiation. Compared to ZH11, the *osg* mutant exhibited decreased grain length and thousand-grain weight but increased grain width and thickness, while displaying significantly reduced plant height, tiller number, pollen viability, and seed setting rate. Map-based cloning revealed that *OsSRS3*, a known regulator of rice grain size and fertility, was identified as the candidate gene for *OSG*. The loss-of-function mutant in *OsSRS3* exhibit abnormal phenotypes similar to that in *osg*. Comparative transcriptome sequencing of young panicles from ZH11 and *osg* showed that up-regulated genes were predominantly enriched in pathways related to plant hormone signal transduction and MAPK signaling, whereas down-regulated genes were mostly associated with starch and sucrose metabolism. Further analysis of differentially expressed genes (DEGs) within the pathways revealed that multiple DEGs in *osg*, such as *OsJAZ11*, *OsUgp1* and *OsUgp2*, have been functionally characterized, and their mutant phenotypes align with those observed in *osg*. These findings provide a crucial foundation for elucidating the molecular mechanisms by which the *SRS3* gene regulates grain size and fertility in rice.

## Introduction

Rice (*Oryza sativa*) is one of the most important food crops, playing a crucial role in agricultural production. Increasing rice yield and quality remains an urgent challenge in rice breeding research [1]. Rice yield is directly influenced by effective panicle number, grains per panicle, and thousand-grain weight [2]. Effective panicle number depends on tiller number, tiller angle, and plant height; grains per panicle depend on panicle length, branches per panicle, and fertility; thousand-grain weight is directly affected by grain shape and size, which also influence the appearance quality of milled rice [3]. Therefore, studying the genetics and molecular mechanisms underlying rice grain shape can aid in genetic improvement of yield and quality traits, which is significant for developing high-yield and high-quality rice varieties.

With the development of functional genomics and molecular biology technologies, an increasing number of grain shape-related genes have been mapped and cloned. Previous studies have shown that rice grain shape is mainly regulated by lemma cell development and involves genetic pathways such as the ubiquitin-proteasome pathway, G-protein signaling pathway, mitogen-activated protein kinase (MAPK) signaling pathway, plant hormone signal transduction pathways, and transcription factor regulation pathways [4]. *Grain Size 3* (*GS3*) was the first cloned grain shape gene, primarily controlling grain length. *GS3* encodes a transmembrane protein with a domain regulating organ size (OSR), which negatively regulates grain length, while the von Willebrand factor type C (VWFC) domain inhibits the effect of OSR on grain length within a certain range [5]. *Grain Width 2* (*GW2*) was the first cloned gene controlling grain width and is the primary regulator of grain width. *GW2* encodes an E3 ubiquitin ligase involved in degrading proteins that promote cell division, thereby controlling grain width [6]. The *seed width on chromosome 5* (*GW5*), located on chromosome 5, is a major quantitative trait locus (QTL) controlling grain width and weight. This gene promotes cell division in the lemma and increases grain width and weight [7,8]. Subsequent studies have found that *GW5* inhibits the activity of transcription factors in the brassinosteroid (BR) signaling pathway, affecting the expression of downstream BR response gene [9]. *Grain Weight 8* (*GW8*) positively regulates grain width by influencing genes related to lemma cell cycle [10]. *Grain Weight 7* (*GW7*), located on chromosome 7, regulates the TONNEAU1 recruitment motif sequence protein and alters grain length-to-width ratio by influencing cell division patterns [11]. *Grain size 2* (*GS2*) regulates grain size by promoting cell division and expansion; *GS2* encodes a growth-regulating factor *OsGRF4* that changes the cell life cycle, thus altering the grain length-to-width ratio [12].

Rice grain shape is closely related to rice yield and quality. Despite the discovery and cloning of numerous QTLs and related genes controlling grain shape, the genetic regulatory network of rice grain shape remains incompletely understood. There is still a need to discover new regulatory genes to fully understand the molecular network regulating grain shape. In this study, we screened for a short-panicle and oval-grain mutant *osg* from the japonica variety ZH11 using radiation mutagenesis. We conducted agronomic trait surveys, histological phenotype analyses, fine mapping to identify the mutated gene, and performed transcriptome analysis between wild-type and *osg*. Our aim is to provide a new theoretical basis for elucidating the molecular network regulating rice grain morphology and to lay an important genetic resource foundation for molecular design breeding in rice.

## Materials and methods

### Experimental materials and growing conditions

The oval-shaped grain mutant *osg* was derived from the japonica variety Zhonghua 11 (ZH11) through ^60^Co-γ ray irradiation mutagenesis. After several generations of selection, a mutant with stable heritable phenotype was obtained. The mutant was crossed with two indica varieties, HJX74 (Hua Jing Xian 74) and 9311, to obtain F_1_ hybrids, and self-crossed to produce F_2_ segregating populations. The plants with the *osg* mutant phenotype were selected from the F_2_ population for gene mapping. All materials were grown in the experimental paddy field at Fengyang Campus, Anhui Science and Technology University (32°52’ 30’‘ N, 117°33’ 15’‘ E) under conventional planting and water-fertilizer management.

### Statistical analysis of major agronomic traits

At maturity, 20 plants each of wild-type ZH11 and mutant *osg* were selected for observation and statistical analysis of plant height and tiller number. Main panicles were harvested to measure panicle length, primary branch number, total grain number per panicle, filled grain number, seed setting rate, grain length, width, and thousand-grain weight. Each group had three biological replicates, with the average value taken from 20 individual plants. Data analysis and graph creation were performed using GraphPad Prism v8.0.2. Paired t-tests were utilized to evaluate the significance of differences between groups.

### Observation and analysis of lemma cells in *osg*

During the heading stage, mature spikelets from mutant *osg* and wild-type ZH11 were selected. Samples were fixed overnight at 4°C in 2.5% glutaraldehyde, dehydrated in a series of ethanol concentrations (30%, 50%, 70%, 85%, 95%, and 100%), replaced with isoamyl acetate, dried at critical point, coated with gold, and observed and photographed under a scanning electron microscope. Cell size and number were calculated using Image J v1.54g.

For paraffin sectioning, samples were fixed in FAA solution (70% ethanol: glacial acetic acid: formaldehyde = 90:5:5) for 48 hours, dehydrated in a series of ethanol concentrations (70%, 85%, 95%, and 100%), and immersed in Paraplast Plus. Sections were cut using a microtome, stained with toluidine blue, and observed under a panoramic high-resolution slide scanning system, with cell sizes measured using Image J v1.54g.

### Observation of fertility in *osg*

Mature panicles from wild-type ZH11 and *osg* were selected during the heading stage. Pollen grains were removed from the anthers and stained with a 1% I_2_-KI solution (0.6% KI, 0.3% I_2_, w/w) for viability analysis. Pollen fertility was observed and photographed using an Eclipse E600561 microscope.

Mature ovules were fixed in FAA solution for 48 hours, washed twice in 50% ethanol, and transferred to 70% ethanol. Adequate numbers of ovules were dissected under a dissection microscope to remove the ovaries, then stained with 2% potassium aluminum sulfate for 20 minutes, followed by staining with 10 mg/L eosin B (dissolved in 4% sucrose solution) for 12 hours and counterstained with 2% potassium aluminum sulfate for 20 minutes. Samples were dehydrated in a series of ethanol concentrations (30%, 50%, 70%, 80%, 90%, and 100%), placed in a 1:1 mixture of absolute ethanol and methyl salicylate for 1 hour, and finally cleared in methyl salicylate for over 1 hour. Observations and photography were conducted using a Leica SP2 laser scanning confocal microscope.

### Fine mapping

Using F_2_ segregating populations obtained by crossing *osg* with HJX74 and 9311, leaf samples from individual plants with the *osg* phenotype were collected, and DNA was extracted using the cetyltrimethylammonium bromide (CTAB) method. InDel markers were designed based on insertions or deletions between ZH11 and HJX74/9311 reference genomes using Primer 3 line website (https://primer3.ut.ee/), synthesized by Universal Biosciences Co., Ltd. (Chuzhou, China)(S1 Table). PCR reactions were performed using a LongGene instrument. Reaction conditions included initial denaturation at 95°C for 15 seconds, annealing at 55°C for 15 seconds, extension at 72°C for 15 seconds, 35 cycles, and final extension at 72°C for 5 minutes. PCR products were analyzed using 4% agarose gel electrophoresis.

In addition, 20 WT and 20 *osg* mutant plants from the F_2_ population were pooled into two pools, and SNP chip detection was performed by Wuhan Shuanglvchuang Biotechnology Research Institute Co., Ltd. using a Green Super Rice 40K high-density rice gene chip containing 44,263 SNPs. Allelic differences between the two pools and their parents were analyzed to locate the genomic segment associated with the phenotype, thereby localizing the *OSG* gene.

### Candidate gene analysis

Based on the results of InDel markers and SNP chip detection, all genes within the mapped interval were annotated using the rice genome annotation database (http://rice.plantbiology.msu.edu/). Functional studies of genes within the interval were reviewed, comparing reported mutant phenotypes to identify candidates with phenotypes similar to *osg*. Exon-intron structures of candidate genes were used to design PCR primers, and PCR product sequencing was used to analyze and verify the sequence of candidate genes in *osg*.

*SRS3* gene loss-of-function mutants were purchased from Bigene Biotech Co., Ltd. (Jiangsu, China). Primers were designed according to target site positions, and PCR product sequencing was used to confirm the sequence mutation status of the *SRS3* gene in T_2_ generation. Agronomic traits such as plant height, tiller number, panicle length, and grain shape were observed in mature plants of *srs3*.

### Transcriptome and qRT-PCR analysis

In the early stage of booting, young panicles (0–3 cm) were collected from ZH11 and *osg*, with three biological replicates of 0.3 g each, including at least five plants. Samples were frozen in liquid nitrogen and sent to Wuhan Biorun Biotechnology Co., Ltd. for RNA-seq (https://plant.biorun.com/. Wuhan, China) using the Illumina NovaSeq 6000 platform. The clean reads were aligned to the rice reference genome (MSU7) using the software Hisat2 with the default parameters [13]. Differential expression analysis was performed using DESeq2, with filtering criteria of Padj < 0.05 and log_2_|Fold Change| > 1. Up-regulated and down-regulated genes were subjected to GO and KEGG enrichment analysis.

For qRT-PCR (quantitative real time PCR), total RNA was extracted from young panicles (0–3 cm) collected from WT and *osg* using TRIzol reagent (Invitrogen), following the manufacturer’s protocols. The total RNA was reverse transcribed into cDNA using the HiScript III RT SuperMix kit (Vazyme, China) according to the instruction manual. The cDNA was diluted and used as the template to perform qRT-PCR with SYBR Green Ⅰ PCR Master Mix system (TOYOBO, Japan). The reaction condition was as follows: 95°C for 2 min, 40 cycles of 95°C for 15 s, 56°C for 15 s, and 72°C for 20 s. The 2^-ΔCT^ method was employed to quantify the relative expression levels of genes. The *Actin* gene in rice was used as an internal control. The gene-specific primers of DEGs were listed in S1 Table.

## Results

### Phenotypic analysis of the *osg* mutant

**Fig 1:**
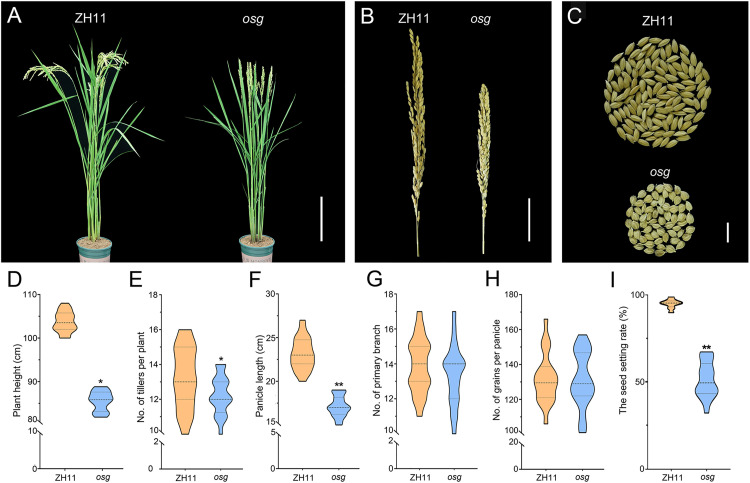
Observation and analysis of plant architecture and panicle morphology in ZH11 and *osg.* **(A)** Observations of plant architecture in ZH11 and *osg* at maturity, scale bar = 20 cm; **(B)** Observations of panicle morphology in ZH11 and *osg* at maturity, scale bar = 5 cm; **(C)** Observations of individual panicle yield in ZH11 and *osg*, scale bar = 1 cm; **(D)** Plant height; **(E)** Tiller number; **(F)** Panicle length; **(G)** Number of primary branches; **(H)** Number of grains per panicle; **(I)** Seed setting rate. n = 20, * indicates P < 0.05; ** indicates P < 0.01. Significance of the results was assessed using a Student’s t-test, two tailed.

To understand the genetic control of rice fertility and grain size, we developed a set of mutants with sterility or/and altered grain appearance. The *osg* mutant was isolated from the M3 generation of the rice cultivar ‘Zhonghua 11’ subjected to mutagenesis with ^60^Co-γ ray irradiation. During the maturation stage of rice, we conducted the agronomic trait survey comparing the wild-type ZH11 with the *osg* mutant. Our analysis revealed that, compared to ZH11, the plant height and tiller number in *osg* were significantly reduced by 27.03% and 7.55%, respectively (Fig 1A, 1D, 1E). Additionally, the panicle length of the *osg* mutant decreased by 25.92%, and the seed setting rate was markedly lower at only 50.2%; however, there was no noticeable difference in the number of primary branches or grains per panicle (Fig 1B, 1C, 1F, 1G, 1H, 1I).

**Fig 2:**
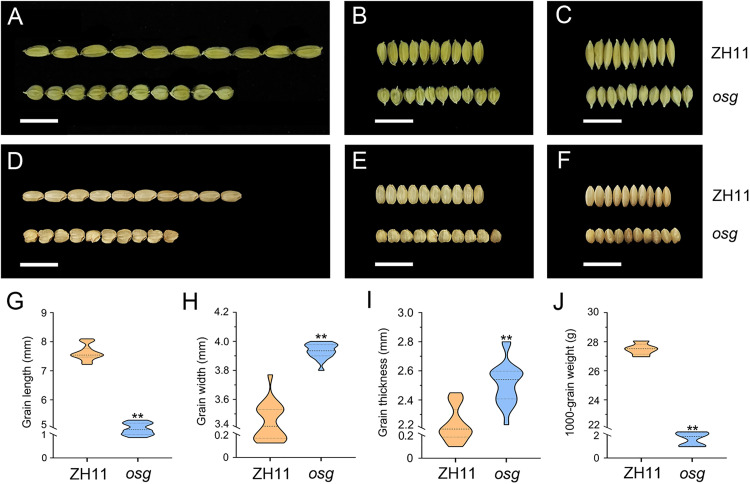
Observation and analysis of grain morphology in ZH11 and *osg.* **(A)** Observations of grain length in ZH11 and *osg*, scale bar = 1 cm; **(B)** Observations of grain width in ZH11 and *osg*, scale bar = 1 cm; **(C)** Observations of grain thickness in ZH11 and *osg*, scale bar = 1 cm; **(D)** Observations of dehulled grain length in ZH11 and *osg*, scale bar = 1 cm; **(E)** Observations of dehulled grain width in ZH11 and *osg*, scale bar = 1 cm; **(F)** Observations of dehulled grain thickness in ZH11 and *osg*, scale bar = 1 cm; **(G)** Grain length in ZH11 and *osg*; **(H)** Grain width in ZH11 and *osg*; **(I)** Grain thickness in ZH11 and *osg*; **J**) Thousand-grain weight in ZH11 and *osg*. n = 20, ** indicates P < 0.01. Significance of the data was assessed using a Student’s t-test, two tailed.

Regarding grain morphology, the grain length in *osg* was significantly shortened by 34.46% compared to ZH11 (Fig 2A, 2D, 2G). Conversely, both grain width and thickness showed significant increases of 14.22% and 12.77%, respectively (Fig 2B, 2C, 2E, 2F, 2H, 2I). Despite these dimensional changes, the thousand-grain weight in *osg* was notably reduced by 22.63% (Fig 2J).

### Observation of caryopsis lemma cells in the *osg* mutant

**Fig 3:**
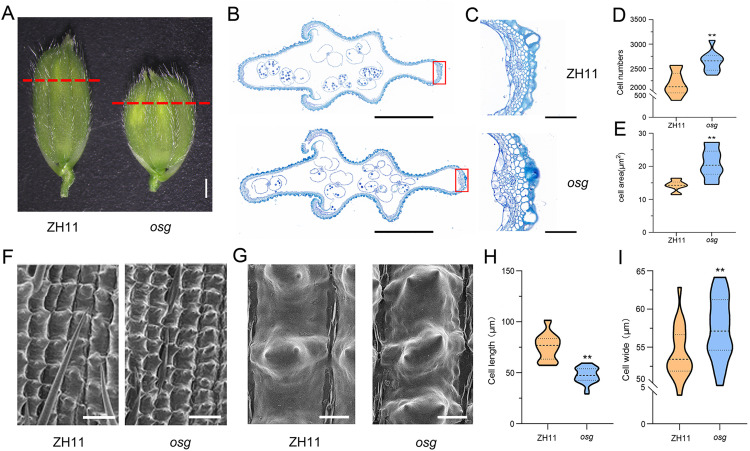
Observation and analysis of caryopsis lemma cells in ZH11 and *osg.* **(A)** Observations of lemmas from ZH11 and *osg*, scale bar = 1 mm; **(B)** Cross-sectional observations of lemmas from ZH11 and *osg*, scale bar = 1 mm; **(C)** Magnified view of lemma cross-sections from ZH11 and *osg*, scale bar = 100 μm; **(D)** Number of cells in the outer parenchyma of the lemma, n = 10; **(E)** Cell area in the outer parenchyma of the lemma, n = 10; **(F)** Scanning electron microscopy (SEM) images of mature caryopsis lemmas from ZH11 and *osg*, scale bar = 100 μm; **(G)** SEM images of mature caryopsis lemmas from ZH11 and *osg*, scale bar = 100 μm; **(H)** Length of outer epidermal cells of the lemma, n = 20; **(I)** Width of outer epidermal cells of the lemma, n = 20. ** indicates P < 0.01. Significance of the data was assessed using a Student’s t-test, two tailed.

Observations of cross-sections of caryopses from both the wild type and the *osg* mutant revealed that, compared to ZH11, the *osg* mutant exhibited an increase in the number of cells within the parenchyma, along with a significant increase in cell area (Fig 3A-3E). Scanning electron microscopy (SEM) observations of the outer epidermis of lemmas from ZH11 and *osg* showed that the contours and arrangement of lemma epidermal cells were clearly distinguishable between ZH11 and *osg* (Fig 3F-3G). Statistical analysis of lemma cell length indicated that the lemma cells in *osg* were significantly shorter than those in ZH11, while the cell width was notably wider (Fig 3H-3I). In summary, the mutation in the *OSG* gene primarily affects the regulation of lemma cell length and width.

### Fertility observation of the *osg* mutant

**Fig 4:**
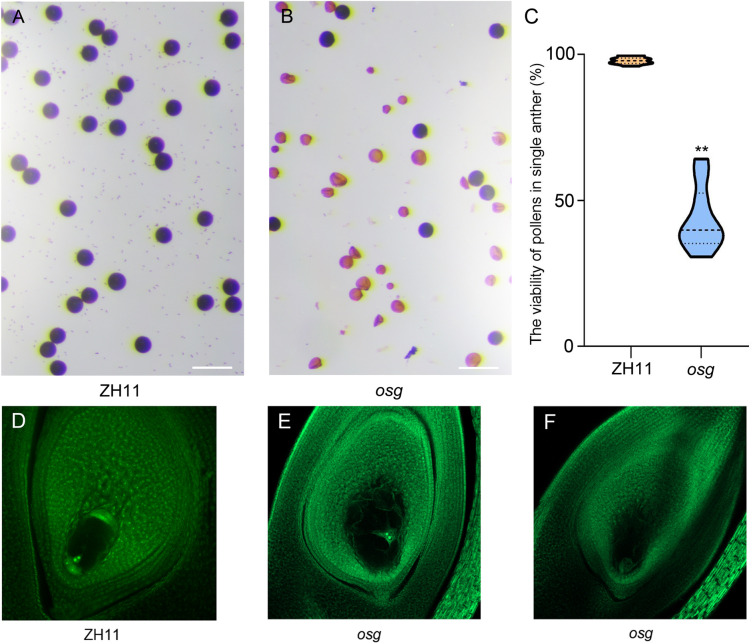
Fertility observation and analysis of the *osg* mutant. **(A)** Staining observation of mature pollen grains from ZH11, scale bar = 100 μm; **(B)** Staining observation of mature pollen grains from *osg*, scale bar = 100 μm; **(C)** Pollen viability comparison between ZH11 and *osg*, n = 10, ** indicates P < 0.01, significance of the results was assessed using a Student’s t-test, two tailed; **(D)** Observation of mature ovules from ZH11; **(E-F)** Observation of mature ovules from *osg*.

In addition to the significant changes in grain morphology, the *osg* mutant exhibited a markedly reduced seed setting rate, with only approximately 50% (Fig 1J). To determine whether the decrease in seed setting rate was associated with pollen fertility, we observed the viability of mature pollen grains from ZH11 and *osg* using a 1% I_2_-KI staining solution. In the wild-type ZH11, pollen grains were plump and stained blackish-brown (Fig 4A), indicating high viability. In contrast, over 50% of the pollen grains from the *osg* mutant appeared yellowish-brown, with only a minority staining blackish-brown (Fig 4B and 4C), suggesting significantly reduced pollen fertility in the *osg* mutant.

To further investigate the fertility of ovules in *osg*, we dissected ovaries from rice plants at maturity and used whole-mount staining and laser scanning confocal microscopy to observe the ovules of both ZH11 and *osg*. Our results showed that, compared to ZH11, some of the ovaries in *osg* developed normally, with clearly visible polar nuclei and embryo sac structures (Fig 4D and 4E). However, in other mature ovules of *osg*, complete embryo sac structures could not be observed (Fig 4D and 4F), indicating abnormal development in some of the ovules within the *osg* mutant, which likely contributes to its reduced fertility and seed setting rate.

### Map-based cloning of the *OSG* gene

**Fig 5:**
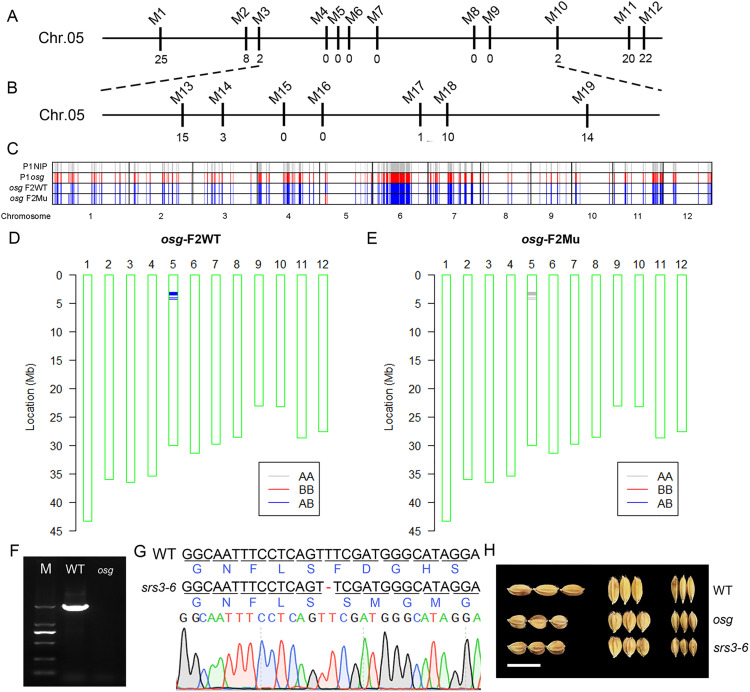
Localization of the *OSG* gene and analysis of candidate genes. **(A)** Preliminary localization of the *OSG* gene using InDel markers M1 and M12 on chromosome 5; **(B)** Fine mapping of the *OSG* gene using newly designed InDel markers between M3 and M10, further narrowed down to between M14 and M17; **(C)** Genotype analysis of parents and F_2_ pools using SNP chip data; parent *osg* appears gray (homozygous), parent NIP appears red (homozygous), indicating homozygous genotypes for both parents; **(D)** SNP analysis at differential loci showing mostly heterozygous genotypes in the WT pool; **(E)** SNP analysis at differential loci showing homozygous mutant genotype (AA) in the mutant pool; **(F)** Amplification abnormalities observed in exons 8, 9, 10 and 11 of the *SRS3* gene in *osg*; **(G)** Identification of a homozygous individual plant *srs3-6* with a single-base deletion (T) leading to loss of function in the *SRS3* gene; **(H)** Observation of grain morphology in *srs3-6*, scale bar = 1 cm.

To localize the *OSG* gene, we utilized an F_2_ population derived from a cross between the *osg* mutant and HJX74. We selected the individual plants with the mutant phenotype and performed linkage analysis using InDel markers designed for the target locus of *osg*. The results indicated that primers M1 and M12 on chromosome 5 were linked to the mutant phenotype of *osg* (Fig 5A). Further InDel markers were designed between M1 and M12 based on the differences in the ZH11 and HJX74 genomes, and the target gene was narrowed down to the region between markers M3 and M10 on chromosome 5. Subsequently, we crossed *osg* with 9311 to obtain another F_2_ population. By designing more densely spaced polymorphic markers within the previously defined interval and analyzing plants with the mutant phenotype, we further narrowed the target region to between M14 and M17 (Fig 5B), corresponding to approximately 3.1 ~ 4.4 Mb region on chromosome 5.

Additionally, we constructed an F_2_ population by crossing *osg* with Nipponbare (NIP) and used SNP chips with bulked segregant analysis (BSA) to locate the *OSG* gene. The SNP chip results showed that the genotype of parent *osg* appeared gray, while that of parent NIP appeared red, indicating homozygous genotypes for both parents (Fig 5C). In the F_2_ segregating population, most genotypes in WT and *osg* mutant pools were heterozygous; however, a segment on chromosome 5 showed differences between the two pools. Comparison of genomic differences revealed that the WT pool was predominantly heterozygous (AB) (Fig 5D), whereas the genotype of the mutant pool matched the *osg* genotype (AA) (Fig 5E). Based on BSA principles and SNPs within this differential segment, the *OSG* gene was limited to approximately 3.1 ~ 3.6 Mb region on chromosome 5.

### Analysis of candidate genes within the precise positioning segment

To identify candidate genes for *osg*, we screened all genes present in the localization region. According to the rice genome annotation database (http://rice.uga.edu/), there are 33 functional genes within the localization interval (S2 Table), among which *SRS3* (LOC_Os05g06280) has been reported to have a mutantion phenotype similar to that of *osg*. To verify whether the *osg* mutant phenotype is due to a mutation in *SRS3*, we amplified and sequenced the exonic regions of the *SRS3* gene in *osg*. The results showed that most exons of *SRS3* in *osg* could be normally amplified and were consistent with the wild-type sequence, except for exons 8, 9, 10, and 11, which failed to amplify (Fig 5F, S1 Fig), indicating an abnormality in the coding sequence of *SRS3* in *osg*.

To further confirm whether the *osg* phenotype is caused by a mutation in *SRS3*, we obtained a functional knockout mutant of *SRS3* and analyzed the target site in 12 individual plants. One of the plants, designated *srs3–6*, was identified as a homozygous mutant with a single-base deletion (T) at the target site, leading to changes in the protein sequence and loss of function of *SRS3* (Fig 5G, S1 Fig). Observations of grain morphology in *srs3–6* showed similarities to *osg*, with significantly shorter grain length and increased grain width and thickness compared to the wild-type ZH11 (Fig 5H). These data collectively suggest that the abnormal phenotype observed in *osg* is due to a sequence variation in *SRS3* (LOC_Os05g06280), thereby identifying LOC_Os05g06280 as the encoding gene for *OSG*.

### Transcriptome analysis of the *osg* mutant

**Fig 6:**
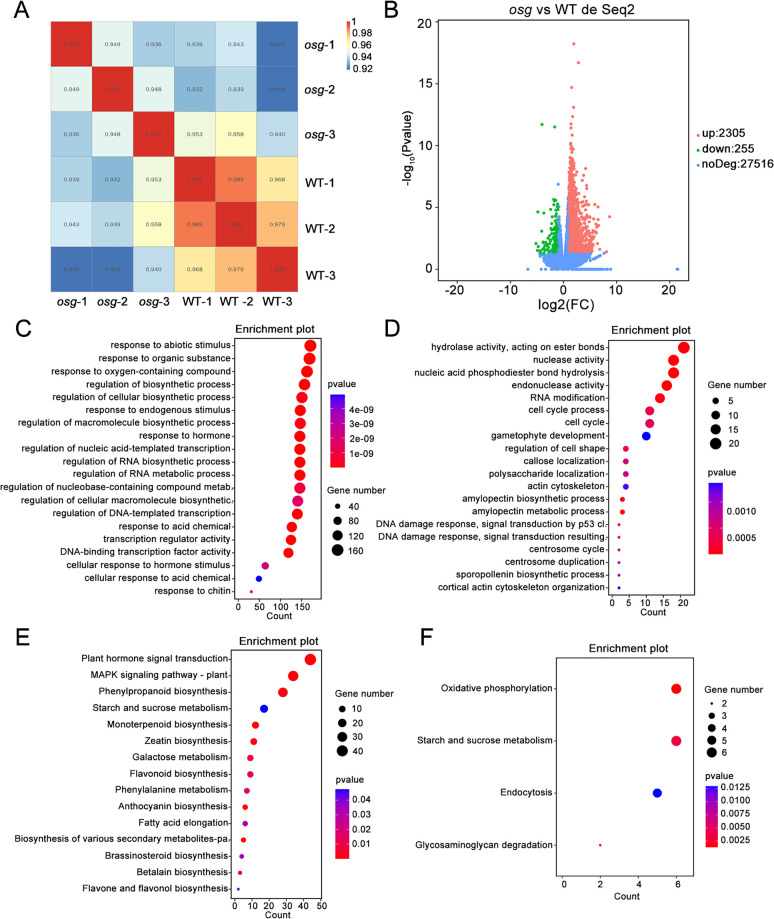
Transcriptome analysis of young panicles in ZH11 and *osg.* **(A)** Correlation analysis between transcriptome samples; **(B)** Volcano plot of differentially expressed genes (DEGs); **(C)** GO enrichment analysis of up-regulated DEGs; **(D)** GO enrichment analysis of down-regulated DEGs; **(E)** KEGG pathway analysis of up-regulated DEGs; **(F)** KEGG pathway analysis of down-regulated DEGs.

To further understand the transcriptomic changes in *osg*, we conducted RNA-Seq analysis on young panicles (0–3 cm) from ZH11 and *osg*. The three biological replicates from ZH11 were named WT-1, WT-2, WT-3, while those from *osg* were named *osg-1*, *osg-2*, *osg-3*, for a total of six samples. The data analysis from the RNA sequencing showed that the correlation coefficients between the wild-type samples and the mutant samples were all above 0.92, indicating satisfactory consistency (Fig 6A). A total of 2560 differentially expressed genes (DEGs) were identified between ZH11 and *osg*, among which 2305 genes were significantly up-regulated and 255 genes were significantly down-regulated in *osg* (Fig 6B), with the number of up-regulated genes notably exceeding that of down-regulated genes (S3 Table, S2 Fig).

GO enrichment analysis of the DEGs revealed that the up-regulated genes were significantly enriched in biological processes such as response to abiotic stimulus, response to organic substance, and response to oxygen-containing compound (Fig 6C). In contrast, the down-regulated genes were significantly enriched in biological processes like hydrolase activity, nuclease activity, and hydrolysis of nucleic acid phosphodiester bonds (Fig 6D).

KEGG pathway analysis indicated that the up-regulated genes were significantly enriched in pathways such as plant hormone signal transduction, MAPK signaling pathway, and phenylpropanoid biosynthesis (Fig 6E, S4 Table, S3-S5 Figs). Meanwhile, the down-regulated genes were significantly enriched in pathways like starch and sucrose metabolism and oxidative phosphorylation (Fig 6F, S5 Table, S6 Fig). These results suggest that the mutation in *OSG* primarily affects pathways related to plant hormone signal transduction, MAPK signaling, and carbohydrate synthesis metabolism (S6-S9 Tables).

### Analysis of differentially expressed genes in KEGG enriched pathways

To further clarify which other genes are affected by the mutation in *OSG* and how these changes lead to the mutant phenotype, we conducted an analysis of pathways enriched with DEGs using the KEGG database.

The up-regulated DEGs were primarily enriched in plant hormone signal transduction pathways with a total of 44 genes, involved including auxin, cytokinin, gibberellin, brassinosteroid (BR), abscisic acid (ABA), ethylene, jasmonic acid (JA), and salicylic acid signaling processes. Through in-depth analysis of these pathways, we found that among the auxin, cytokinin, abscisic acid, and ethylene signaling pathways, 12, 3, 6, and 6 genes were significantly up-regulated respectively, with most genes showing an increase of more than 2.5-fold (S6 Table, S3 and S4 Figs). In the gibberellin signaling pathway, a total of 12 genes were significantly up-regulated, involving the GID, DELLA, and PIF3 families. In the gibberellin signaling pathway, a total of 12 genes were up-regulated, involving the GID, DELLA, and PIF3 families. Among these, 8 genes belong to the GID family, 3 genes belong to the DELLA family, and 1 gene belongs to the PIF3 family. Specifically, *Os07g44890*, *SLRL1* (LOC_Os01g45860), and *OsPIL16* (LOC_Os05g04740) showed significant up-regulation in the mutant compared to ZH11, with more than a 2-fold increase. It has been reported that the *OsPIL16* acts as a negative regulator of grain length in rice, and silencing of *OsPIL16* increases the grain length [14]. This suggests that *OSG* genes may co-participate with *OsPIL16* in the regulation of rice grain length.

**Fig 7:**
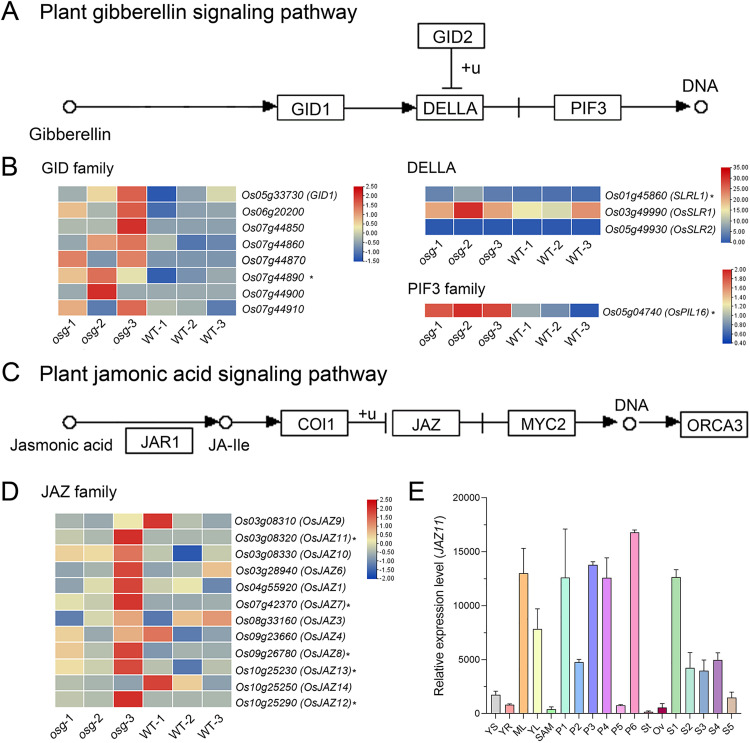
Significantly up-regulated genes in gibberellin and jasmonic acid signaling pathways in *osg.* **(A)** Schematic of the gibberellin signaling pathway; **(B)** Analysis of differentially expressed genes in the gibberellin signaling pathway; **(C)** Schematic of the jasmonic acid signaling pathway; **(D)** Analysis of differentially expressed genes in the jasmonic acid signaling pathway; **(E)** Expression analysis of *OsJAZ11* at various stages of rice development. * indicates up-regulated DEGs.

In the jasmonic acid signaling pathway, several families are involved in signal transduction, including the JAR1 family, COI1 family, JAZ family, and MYC2 family. Analysis revealed that 12 genes were up-regulated, all belonging to the JAZ family. These include *OsJAZ9* (LOC_Os03g08310), *OsJAZ11* (LOC_Os03g08320), *OsJAZ10* (LOC_Os03g08330), *OsJAZ6* (LOC_Os03g28940), *OsJAZ1* (LOC_Os04g55920), *OsJAZ7* (LOC_Os07g42370), *OsJAZ3* (LOC_Os08g33160), *OsJAZ4* (LOC_Os09g23660), *OsJAZ8* (LOC_Os09g26780), *OsJAZ13* (LOC_Os10g25230), *OsJAZ14* (LOC_Os10g25250), and *OsJAZ12* (LOC_Os10g25290). Compared to ZH11, these genes showed varying levels of increased expression in *osg*, with *OsJAZ11*, *OsJAZ7*, *OsJAZ8*, *OsJAZ13*, and *OsJAZ12* being significantly up-regulated by more than 2.5-fold (Fig 7C and 7D). Expression analysis indicated that *OsJAZ11* is preferentially expressed at multiple stages during rice panicle development (Fig 7E). Previous studies have shown that overexpression of *OsJAZ11* leads to reduced panicle length and increased grain width in rice plants, resulting in significant decreases in yield and seed setting rate [15]. These findings suggest that *OSG* genes may co-regulate rice grain width, panicle length, and seed setting rate together with the *JAZ11* gene within the jasmonic acid signaling pathway.

**Fig 8:**
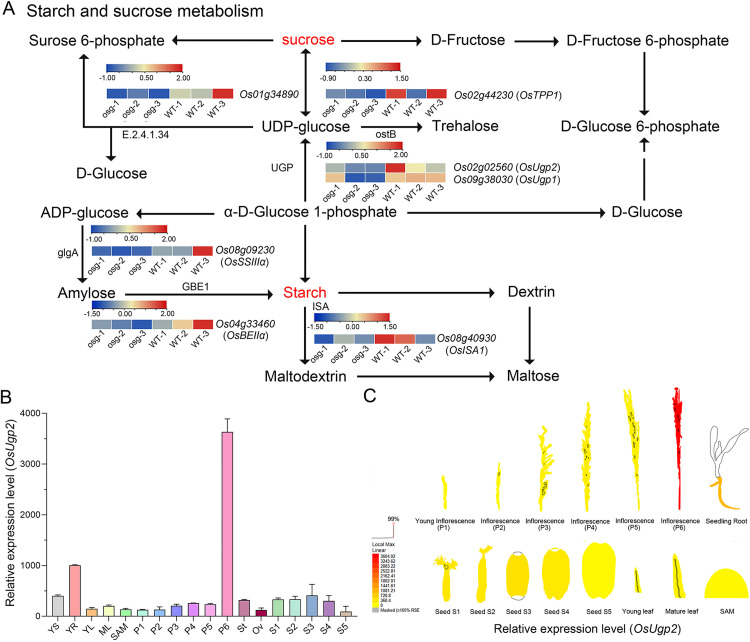
Significantly down-regulated genes in the starch and sucrose metabolism pathway in *osg.* **(A)** Analysis of differentially expressed genes in the gibberellin signaling pathway; **(B-C)** Expression analysis of the *OsUgp2* gene at various stages of rice development. In Panel B, YS indicates 7-day-old Seedling, YR indicates Root of a 7-day-old Seedling, YL indicates young leaf, ML represents Mature leaf, SAM, P1-P6 represent inflorescence (P1, up to 3 cm) (P2, 3-5 cm) (P3, 5-10 cm) (P4, 10-15 cm) (P5, 15-22 cm) (P6, 22-30 cm), St indicates stigma, Ov represents Ovary, S1-S5 indicate Seed (S1, 0-2 dap) (S2, 3-4 dap) (S3, 5-10 dap) (S4, 11-20 dap) (S5, 21-29 dap). * indicates down-regulated DEGs.

The down-regulated differentially expressed genes are mainly enriched in the oxidative phosphorylation pathway and the starch and sucrose metabolism pathway (S8 and S9 Tables). More down-regulated genes are enriched in the starch and sucrose metabolism pathway, with a total of 7 genes showing decreased expression in this pathway (S8 Table). These genes include *Os01g34890*, *OsTPP1* (LOC_Os02g44230), *OsUgp1* (LOC_Os09g38030), *OsUgp2* (LOC_Os02g02560), *OsSSSⅢα* (LOC_Os08g09230), *OsBEⅡα* (LOC_Os04g33460), and *OsISA1* (LOC_Os08g40930). Compared to ZH11, most of these genes showed reduced expression levels in *osg*, decreasing to half or even lower (Fig 8A). These genes play crucial roles in plant carbohydrate metabolism. Expression analysis revealed that *OsUgp2* is specifically expressed during the formation stage of mature rice pollen (P6) (Fig 8B and 8C). Previous studies have shown that silencing of *OsUgp1* and *OsUgp2* leads to pollen abortion in rice [16–18], suggesting that *OSG* gene might regulate rice pollen development by controlling the expression of *OsUgp1* and *OsUgp2*.

To confirm the results of RNA-Seq analysis, we selected several up-regulated or down-regulated DEGs for qRT-PCR analysis on young panicles (0–3 cm). The results showed that four genes (*SLRL1*, *OsPIL16*, *JAZ10*, *JAZ11*) were significantly up-regulated and two genes (*OsUgp1*, and *OsUgp2*) were significantly down-regulated in the *osg* mutant (S7 Fig), which were consistent with the transcriptomic results.

## Discussion

### *SRS3* gene involvement in rice fertility and grain development

Grain size is a crucial agronomic trait that determines rice yield and is an important target for genetic improvement in high-yield breeding. Therefore, identifying grain-size-related mutants in rice and elucidating the regulatory mechanisms of grain size are of great significance for high-yield rice breeding. This study obtained a mutant *osg* with stable inheritable phenotypes through mutation induced by ^60^Co-γ rays. Observaion of agricultural traits on *osg* revealed significantly reduced plant height and tiller number; notably shorter panicle length, no significant changes in primary branch number or grains per panicle, but a significant decrease in seed setting rate due to markedly reduced pollen viability. Regarding grain morphology, the grain length of *osg* was significantly reduced while grain width and thickness were significantly increased, leading to a significant decrease in thousand-grain weight.

Through map-based cloning, the *OSG* gene was localized to the 3.1–3.6 Mb (500kb) region on chromosome 5 (Fig 5A-5E). Analysis of all genes within this interval found that the coding region of the *SRS3* gene could not be normally amplified in the *osg* mutant, and this was verified through the identification and analysis of gene-edited mutants of *SRS3* (Fig 5F-5H). Studies have shown that the *SRS3* gene is a member of the Kinesin 13 subfamily, encoding a protein composed of 819 amino acid residues with a motor domain and a coiled-coil structure [19]. The *srs3* mutant exhibits reduced plant height, shortened panicle length, significantly decreased grain length, and significantly increased grain width and thickness. This gene regulates grain length by controlling lemma cell length [19–21]. These results indicate that the sequence variation of the *SRS3* gene in the *osg* mutant led to its phenotypic variation, confirming *SRS3* as the gene encoded by *OSG*.

These data showed that the loss of function of the *SRS3* gene led to developmental abnormalities in rice plant height, panicle length, pollen fertility, and grain morphology, indicating that the *SRS3* gene plays a critical regulatory role in these processes. However, the downstream regulated genes and molecular regulatory mechanisms remain unclear.

### Function of kinesins in plant growth and development

The Kinesin-13 family comprises microtubule-binding proteins containing a conserved kinesin motor domain in their mid-region amino acid sequences. Kinesin-13s are motor proteins involved in microtubule (MT) depolymerization, which is crucial for regulating MT dynamics during mitosis, particularly chromosome segregation [22]. Kinesin-13A and Kinesin-13B are two types of internal-motor kinesins [23]. Kinesin-13A colocalizes with Golgi bodies in Arabidopsis cells, suggesting it is a special type of internal-motor kinesin [24]. In Arabidopsis, the Kinesin-13A protein preferentially localizes to cortical microtubules in secondary cell walls, preventing cell wall deposition via MT depolymerization, which is essential for the establishment of secondary cell walls in Arabidopsis [25]. In rice, OsKinesin13A is an active MT-depolymerizing enzyme mainly distributed in vesicles derived from the Golgi apparatus and localized on microtubules. Mutations in OsKinesin13A lead to poorly developed plants with smaller grains due to altered MT orientation, resulting in changed cellulose microfibril direction and cell elongation [20–22]. Research has also shown that *BHS1* (*SRS3*) encodes the kinesin Kinesin-13A, which regulates grain length downstream of BRI, negatively regulating BR signaling and rice architecture [26].

In this study, the identified *osg* is a novel allele variant of *SRS3*. The mutation in *OSG* leads to aberrant coding of the motor protein Kinesin-13A, resulting in shortened grain length and increased grain width in rice. Combined with RNA-seq analysis, it was found that the abnormal expression of the *OSG* gene caused significant changes in the expression of numerous other genes, affecting pathways such as auxin signaling, gibberellin signaling, jasmonic acid signaling, and brassinosteroid (BR) signaling. These findings provide theoretical insights into the molecular regulatory network of Kinesin-13A in rice.

### Possible mechanisms of *SRS3* regulation on rice grain size and fertility

RNA-seq analysis showed that mutations in the *OSG* gene resulted in differential expression changes in several genes. Most up-regulated genes were enriched in pathways such as plant hormone signal transduction, while most down-regulated genes were enriched in starch and sucrose metabolism pathways.

Plant hormone signal transduction participates in almost all stages of plant growth and development. As the main component of rice yield, rice grains are influenced by multiple hormones during their formation and development [27]. Analysis of differentially expressed genes revealed that the *OsPIL16* gene was up-regulated in gibberellin signaling pathways. Previous studies indicate that APG, encoded by this gene, acts as an antagonistic interaction factor of PGL1, inhibiting the increase in rice grain size by suppressing the expression of the *PGL1* gene [14]. Additionally, multiple JAZ family genes in the jasmonic acid signaling pathway were up-regulated in the *osg* mutant, including *OsJAZ11* and *OsJAZ10*. According to previous research, *OsJAZ11* coordinates the expression of *OsGW7* and MADS genes, regulating grain size and floret development [28,29], whereas overexpression of *OsJAZ10* affects grain width and thickness [30].

Sucrose metabolism plays a key role in plant development, stress response, and yield formation. The synthesis and degradation of sugars drive the entire process of plant growth and development, with sucrose acting as a signaling molecule that regulates related gene expression and interacts with other genes, hormones, and defense signals [31]. Through the analysis of down-regulated genes enriched in the starch and sucrose metabolism pathway, we identified *OsUgp2*, primarily expressed in pollen and developmentally regulated. Silencing of *OsUgp2* leads to the inability to form mature pollen, and pollen fertility correlates with the amount of *OsUgp2* mRNA [17], suggesting that the reduced pollen fertility in the *osg* mutant may be associated with the down-regulation of *OsUgp2* expression.

In summary, we speculate that the mutation in *SRS3* affects multiple genes such as *OsPIL16*, *OsJAZ11*, *OsJAZ10*, and *OsUgp2* involved in plant hormone signal transduction and starch and sucrose metabolism pathways, thereby collectively influencing the plant height, grain morphology, pollen fertility, and other traits of the *osg* mutant. These findings help understand the interrelationships between metabolic pathways and pathways during rice grain development and provide theoretical references for further analyzing and confirming downstream regulated genes of *OSG*, offering insights into the molecular regulatory networks of rice grain morphology and pollen fertility.

## Supporting information

S1 Fig Primer design of the *SRS3* gene and mutant identification. ATG and TAG represent the start codon and stop codon, respectively. The arrows (F1-18, R1-18) indicate the location of the primers for gene sequence analysis. The red vertical line represents the target site of gene editing. The primers of F9 and R9 were used for identifying the CRISPR/Cas9-mediated mutations in SRS3 in the T2 generation. The black letters indicate the DNA sequences with WT in the target site. The sequencing peak plot of the target site sequence is represented by the curve. The dotted lines indicate nucleotide deletions, and dark blue letters indicate the amino acids, and the asterisk (*) represents the stop codon generated in advance.
(TIF)

S2 Fig RNA-Seq analysis between ZH11 and *osg.* (A) Gene expression analysis of ZH11 and *osg* samples; (B) Heat map of differentially expressed genes (DEG); (C-D) Some pathway classes in which the differentially expressed up-regulated (C) and down-regulated (D) genes were significantly enriched, respectively.
(TIF)

S3 Fig The heatmap of up-regulated genes in the auxin signaling pathway. (A) Auxin signal transduction pathway diagram; (B-F) Heat map of up-regulated DEGs from AUX (C), ARF (C), IAA (D), GH3 (E), SAUR (F) family in RNA-seq between *osg* and ZH11.
(TIF)

S4 Fig The heatmap of up-regulated genes in the cytokinine, ehtylene, brassinosteroid, abscisic acid, salicylic acid signaling pathway. (A-E) The diagram of biosynthesis or signal transduction pathway and heat map of zeatin biosynthesis (A), cysteine and methionine metabolism (B), brassinosteroid biosyuthesis (C), ABA signaling (D), and salicylic acid signaling pathway (E).
(TIF)

S5 Fig The heatmap of up-regulated genes in the MAPK signaling pathway. (A) The diagram of MAPK signal transduction pathway in plants; (B-G) Heat map of up-regulated DEGs from EPF/EPFLs (B), SERKs (C), TMM (D), ER/ERL (E), MAP3Ks (F), MAP2Ks (G), MAPKs (H) families, respectively.
(TIF)

S6 Fig The heatmap of down-regulated DEGs in the oxidative phosphorylation pathway. (A) The diagram of oxidative phosphorylation pathway process; (B-D) Heat map of down-regulated DEGs for NADH dehydrogenase (B), cytochrome c oxidase (C), ATP synthesis (D), respectively.
(TIF)

S7 Fig qRT-PCR analysis of DEGs in WT and *osg*. The samples for qRT-PCR analysis are young panicles from WT and *osg*; Values are means ± SD (standard deviations) of three independent biological replicates. Two asterisks (**, P < 0.01) indicate extremely significant differences between WT and *osg* lines as determined by Student’s t-test.
(TIF)

S1 Table Primer design in this study. (XLSX)

S2 Table Screening of candidate genes in the localization region. (XLSX)

S3 Table Down and Up-regulated DEGs between *osg* and ZH11 in RNA-Seq analysis. (XLSX)

S4 Table KEGG enrichment pathways of up-regulated DEGs in *osg* vs ZH11. (XLSX)

S5 Table KEGG enrichment pathways of down-regulated DEGs in *osg* vs ZH11. (XLSX)

S6 Table Up-regulated genes in the plant hormone signaling pathway. (XLSX)

S7 Table Up-regulated Genes in the MAPK signaling pathway. (XLSX)

S8 Table Down DEGs in the starch and sucrose metabolism pathway. (XLSX)

S9 Table Down DEGs in the oxidative phosphorylation pathway. (XLSX)
