# Supplementary figures and images for: Fine mapping and transcriptomics reveal OSG function in regulation of grain size and pollen fertility in rice (Oryza sativa)
Source: PLoS One. 2026 Jan 8;21(1):e0338401. doi: 10.1371/journal.pone.0338401 (PMC12782402; doi:10.1371/journal.pone.0338401)

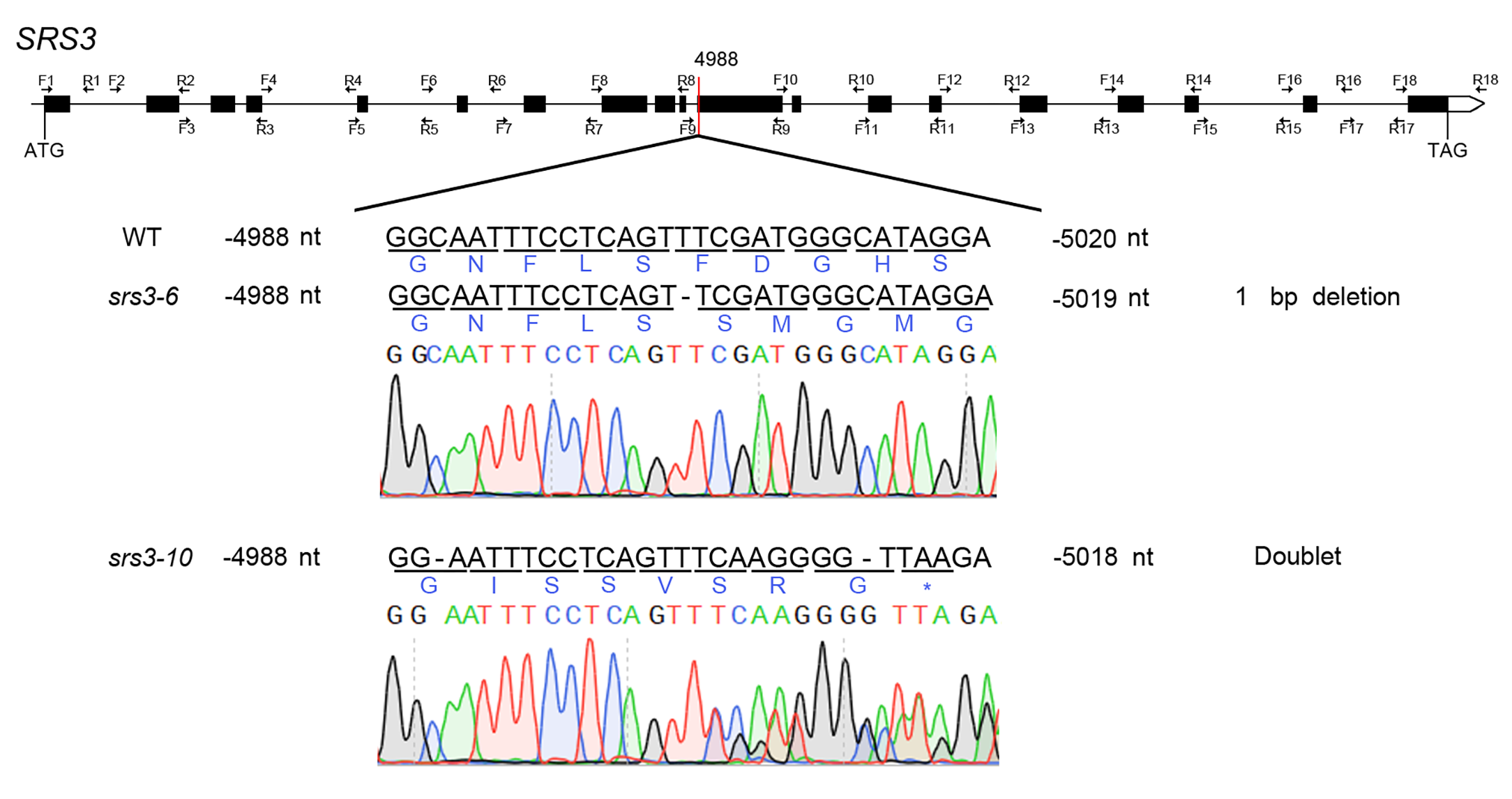

Supplement: S1 Fig — ATG and TAG represent the start codon and stop codon, respectively. The arrows (F1-18, R1-18) indicate the location of the primers for gene sequence analysis. The red vertical line represents the target site of gene editing. The primers of F9 and R9 were used for identifying the CRISPR/Cas9-mediated mutations in SRS3 in the T2 generation. The black letters indicate the DNA sequences with WT in the target site. The sequencing peak plot of the target site sequence is represented by the curve. The dotted lines indicate nucleotide deletions, and dark blue letters indicate the amino acids, and the asterisk (*) represents the stop codon generated in advance. (TIF) [file pone.0338401.s001.tif]

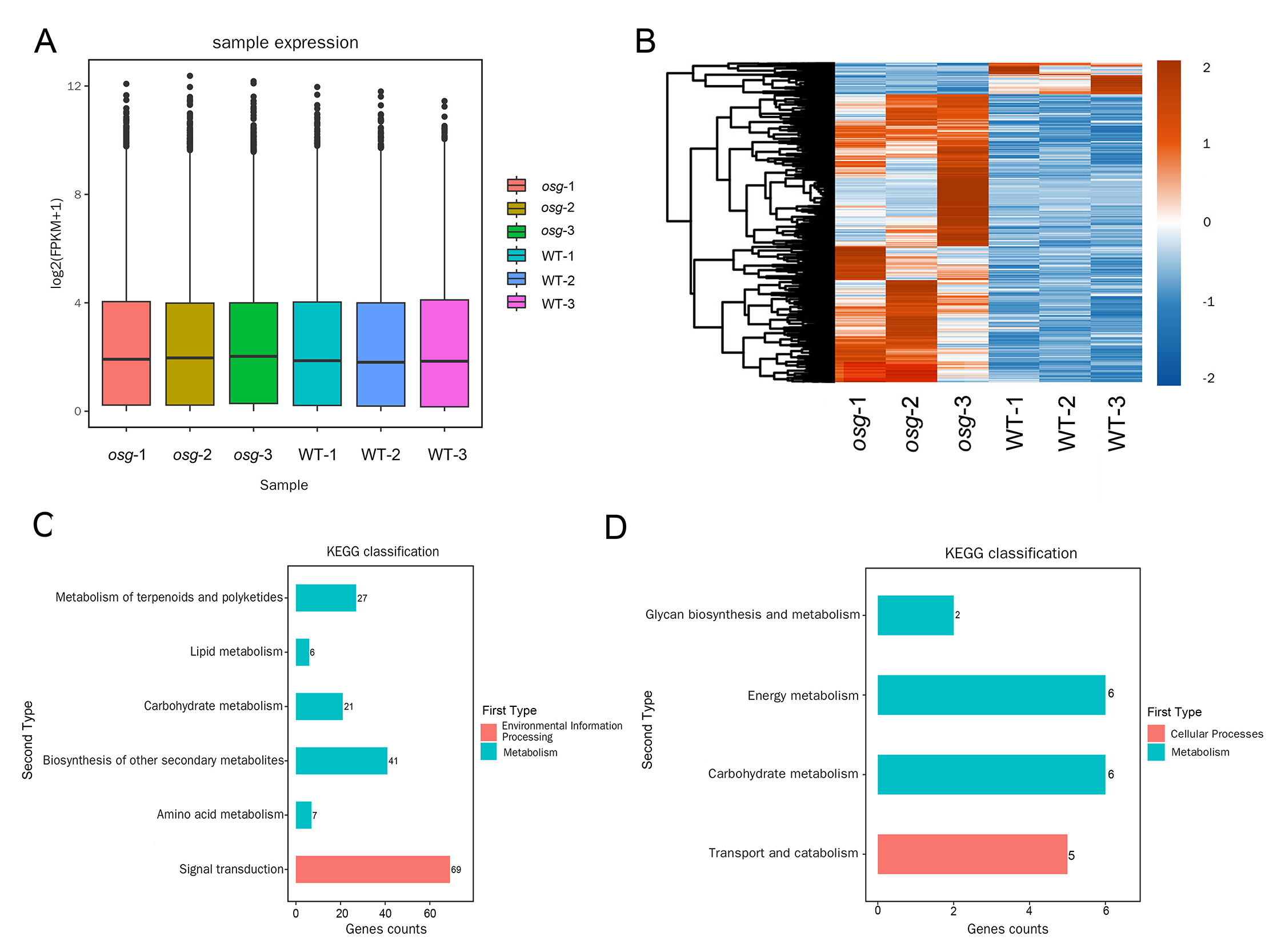

Supplement: S2 Fig — (A) Gene expression analysis of ZH11 and osg samples; (B) Heat map of differentially expressed genes (DEG); (C-D) Some pathway classes in which the differentially expressed up-regulated (C) and down-regulated (D) genes were significantly enriched, respectively. (TIF) [file pone.0338401.s002.tif]

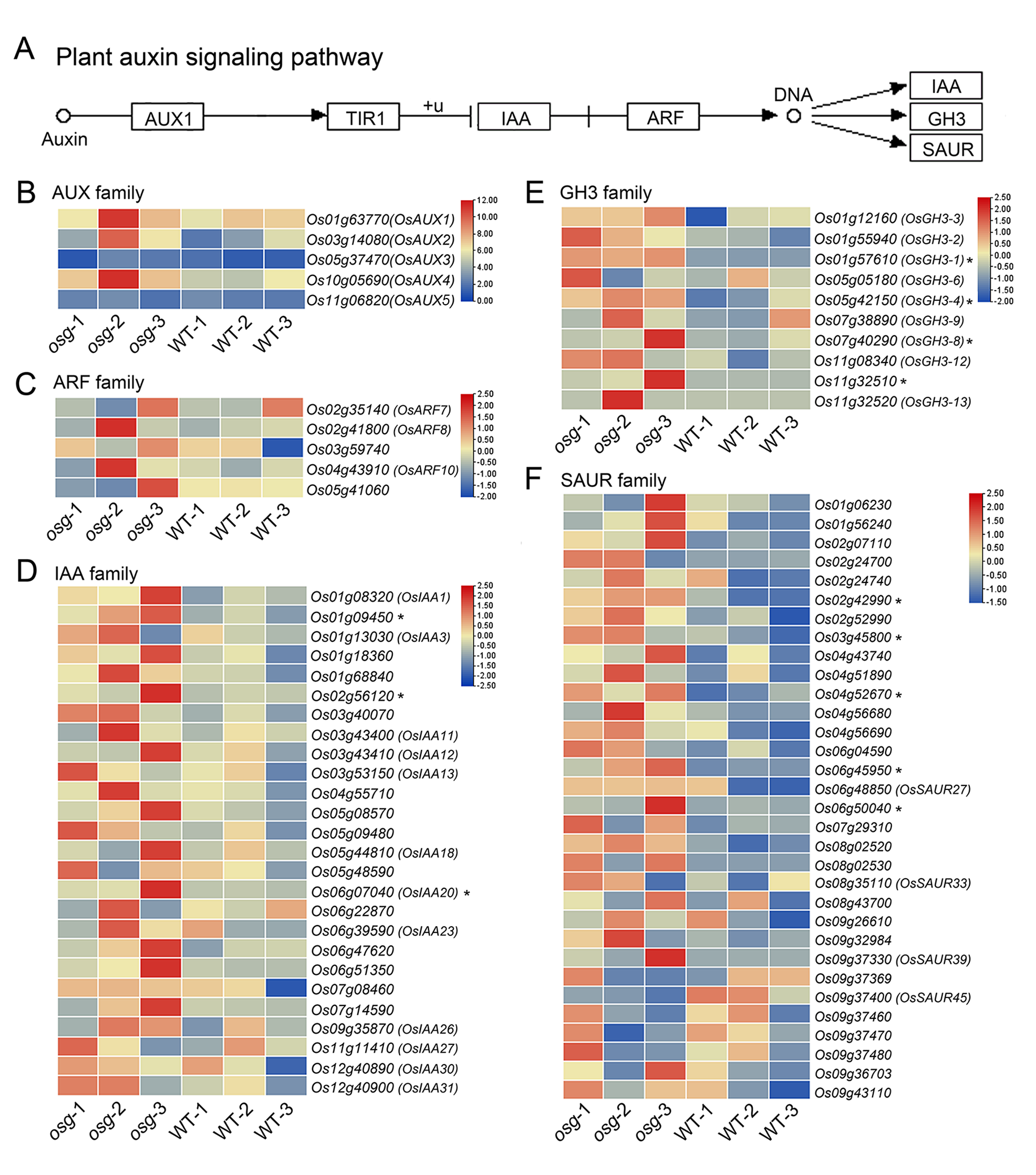

Supplement: S3 Fig — (A) Auxin signal transduction pathway diagram; (B-F) Heat map of up-regulated DEGs from AUX (C), ARF (C), IAA (D), GH3 (E), SAUR (F) family in RNA-seq between osg and ZH11. (TIF) [file pone.0338401.s003.tif]

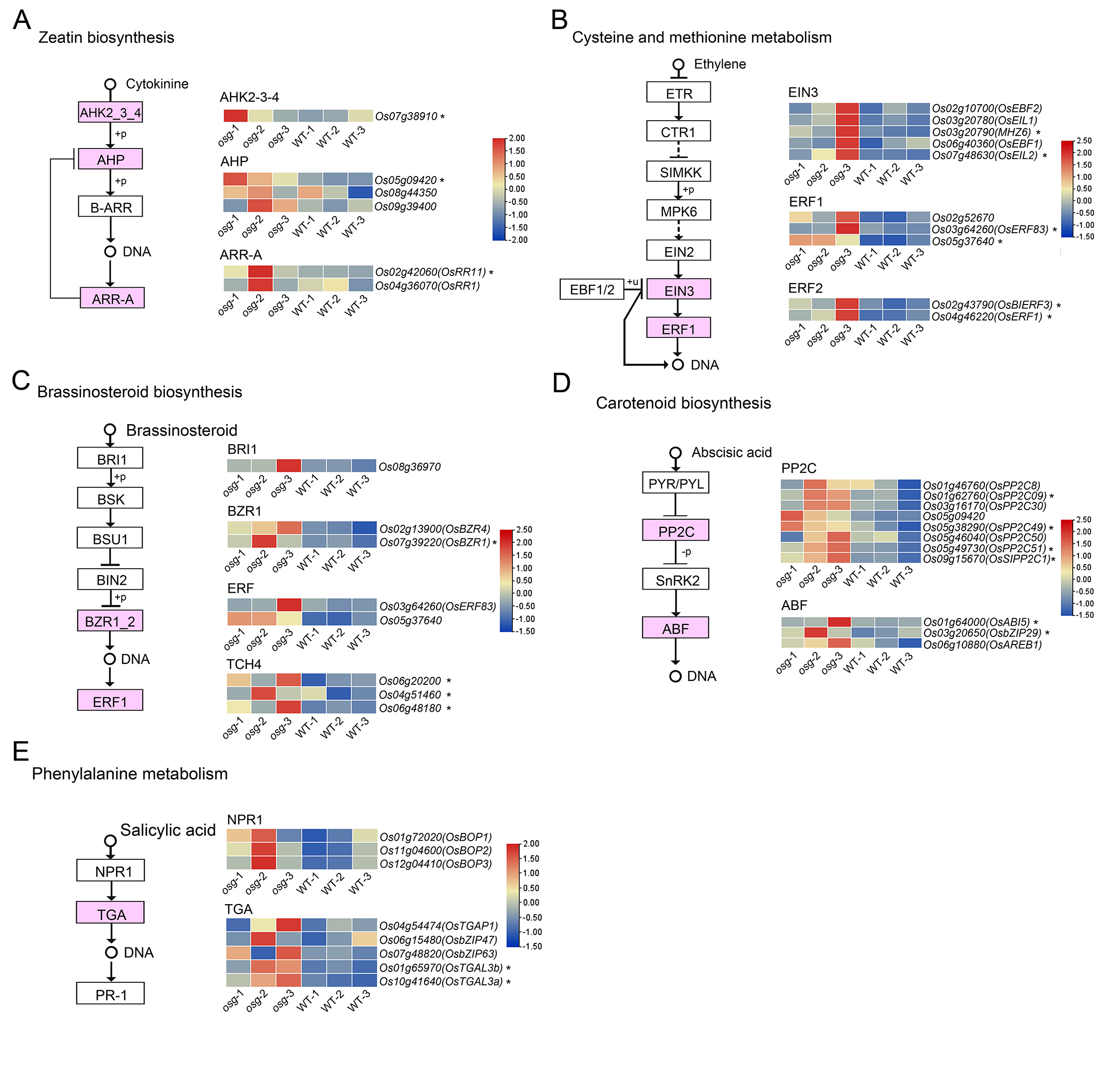

Supplement: S4 Fig — (A-E) The diagram of biosynthesis or signal transduction pathway and heat map of zeatin biosynthesis (A), cysteine and methionine metabolism (B), brassinosteroid biosyuthesis (C), ABA signaling (D), and salicylic acid signaling pathway (E). (TIF) [file pone.0338401.s004.tif]

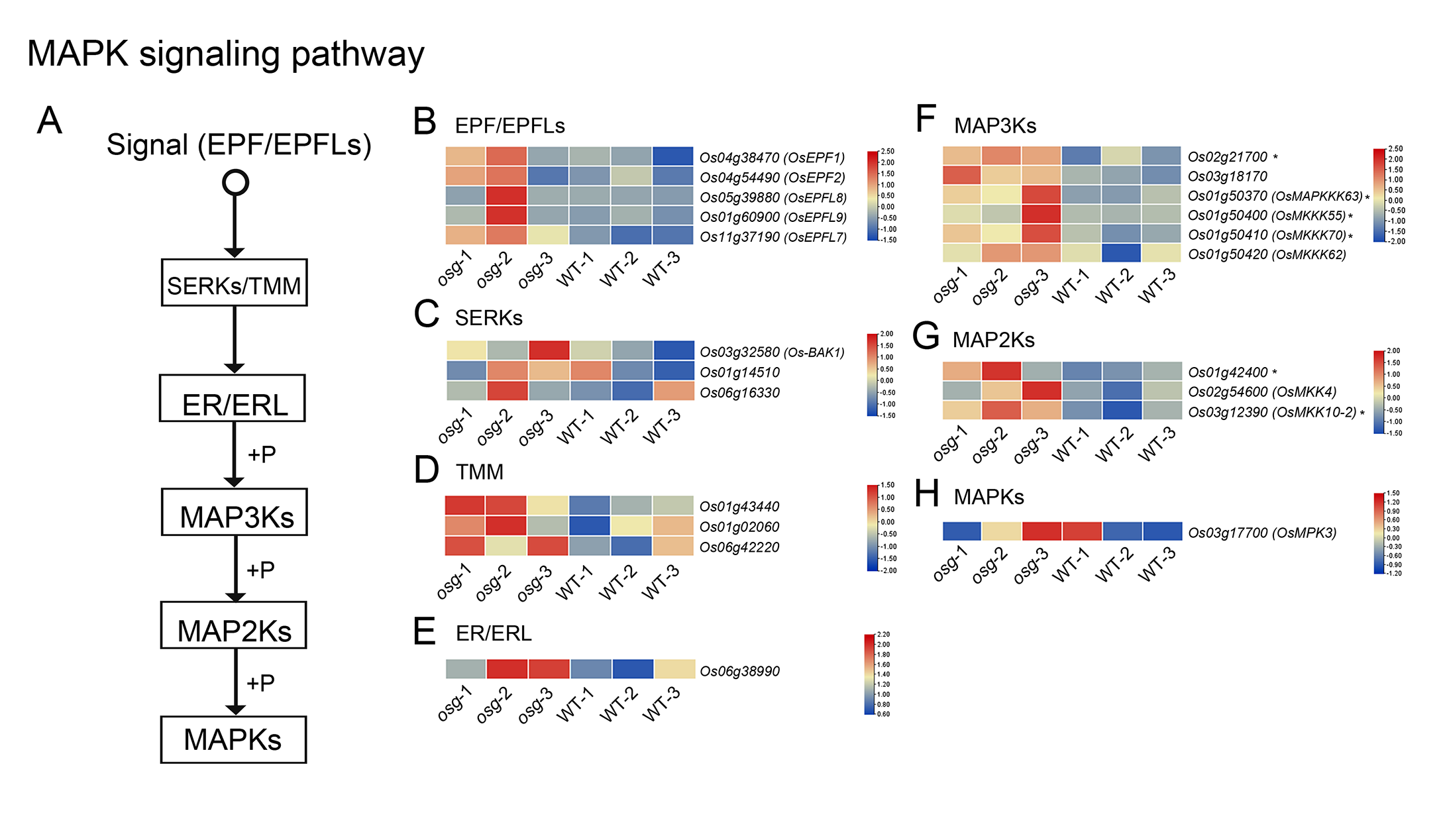

Supplement: S5 Fig — (A) The diagram of MAPK signal transduction pathway in plants; (B-G) Heat map of up-regulated DEGs from EPF/EPFLs (B), SERKs (C), TMM (D), ER/ERL (E), MAP3Ks (F), MAP2Ks (G), MAPKs (H) families, respectively. (TIF) [file pone.0338401.s005.tif]

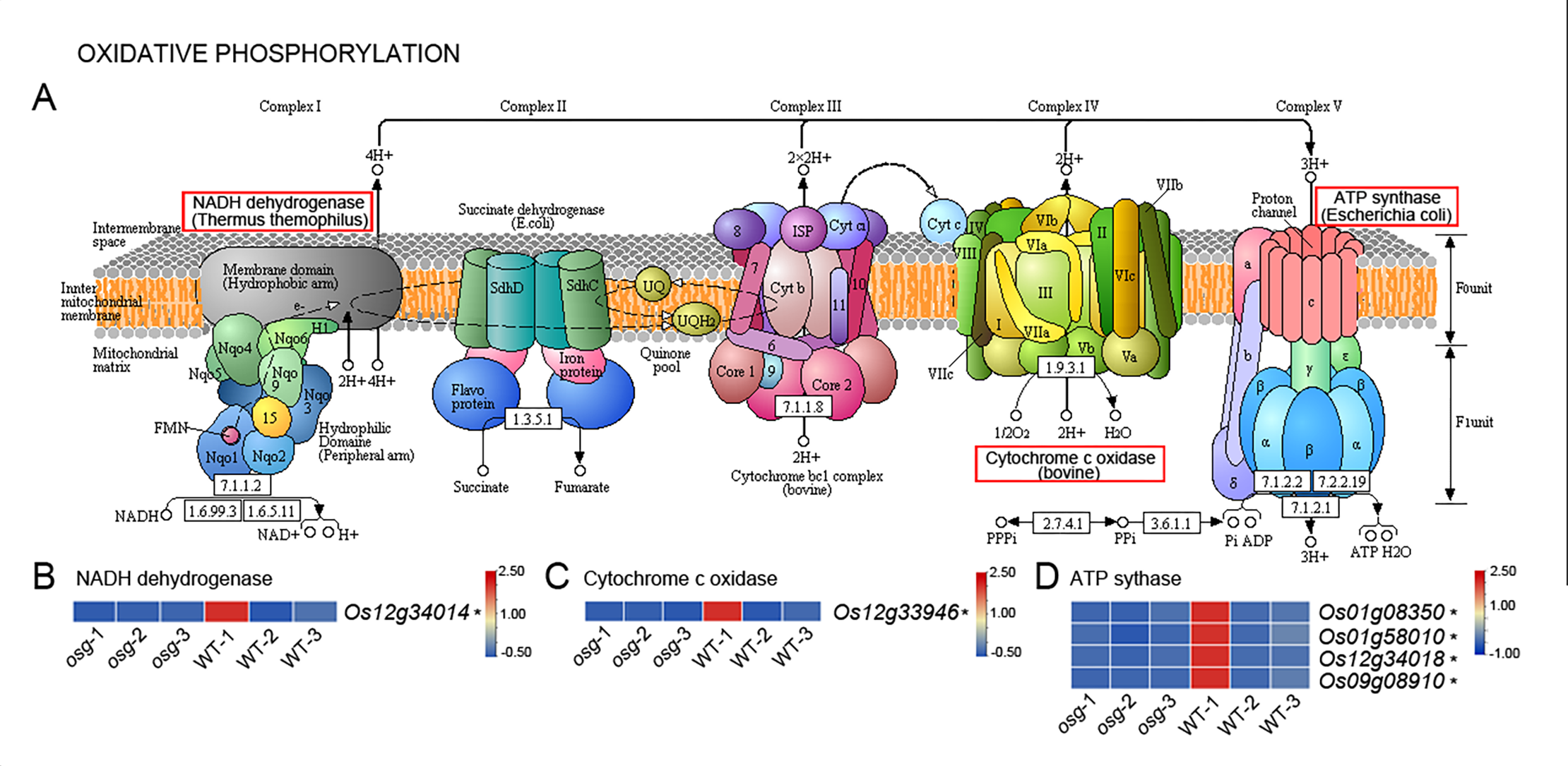

Supplement: S6 Fig — (A) The diagram of oxidative phosphorylation pathway process; (B-D) Heat map of down-regulated DEGs for NADH dehydrogenase (B), cytochrome c oxidase (C), ATP synthesis (D), respectively. (TIF) [file pone.0338401.s006.tif]

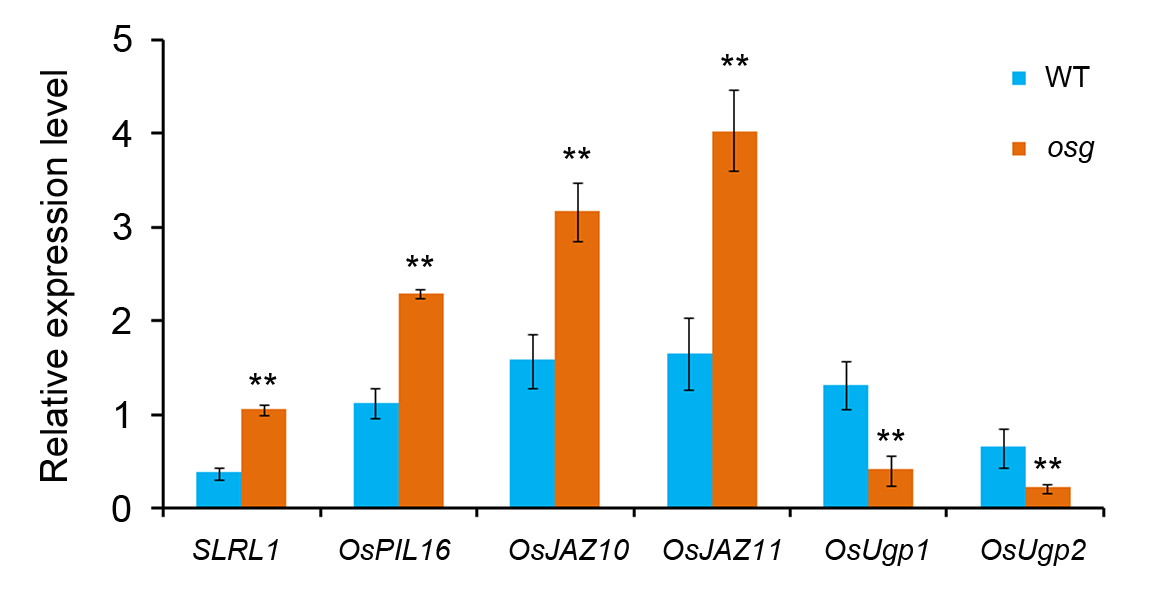

Supplement: S7 Fig — Two asterisks (**, P < 0.01) indicate extremely significant differences between WT and osg lines as determined by Student’s t-test. (TIF) [file pone.0338401.s007.tif]
